# Selections

**Published:** 1893-01

**Authors:** 


### *SELECTIONS.*

---

### The Question of Early High Amputation in Senile Gangrene.

Dr. Powers reports a case to emphasize the importance of amputation through the thigh where senile gangrene has extended from the toes to the foot.

The patient was sixty-seven years old, and had suffered with syphilis in early manhood, but had had no later manifestations. He had used alcoholics moderately. For several months he had had indefinite pains in leg and foot, slight at first, but later becoming so severe as to prevent locomotion. Areas of diminished and lost sensation had in turn appeared. When he came under observation the entire foot was without surface-sensibility —livid and cold. Its dorsum was covered with occasional blebs. The leg, as far up as its middle, showed scattered areas of insensibility and patches of lightish purple mottling, there being, however, no distinct line of demarkation. No evidence of recent or old injury to the toes or foot could be found.

There was no pulsation to be felt in either of the tibials, in the popliteal, or in the femoral at Hunter's canal. The latter vessel pulsated under Poupart's ligament. The heart sounds were weak but otherwise normal. The urine was negative upon examination.

The continuous pain had tended to weaken the patient's general strength ; he had developed a slight daily fever.

After consulting with Dr. Wm. T. Bull, he amputated through the middle of the thigh by anterior-posterior flaps. The femoral artery was thickened, calcareous, and occluded by a firm clot. It was secured by heavy cat-gut, and did not pulsate after removal of the bandage which had been lightly placed about the thigh at the upper third. Occlusion did not extend to the other vessels of the stump.

The operation was followed by no shock, but the patient developed hypostatic pneumonia and died at the end of the third day. The flaps, however, showed primary union with no areas of malnutrition and no pus.

Dr, Powers refers to the writing of Jonathan Hutchinson, who believed that the amputation for senile gangrene due to arterio-sclerosis was only followed by sloughing of the flaps when the part was removed too near the disease. When amputation is done at a low point, the condition of the vessels will rarely be found to be such as to admit of repair ; gangrene of the stump usually occurs immediately, and places the patient's life in much more danger than before the operation.

He urges amputation above the knee where there is gangrene of the foot, and near the shoulder-joint where there is gangrene of the hand. He adduces a number of cases in which he successfully amputated through the lower third of the thigh for gangrene of the foot.

Heidenhain published cases of senile gangrene of the lower extremity which he has seen in the clinic of Küster at the Augusta Hospital in Berlin, and states that Küster had at first contented himself with the simpler form of interference—low amputation. The constant occurrence of gangrene in the amputation-wound, however regularly, compelled further high amputation, so that he was led through his practical experience to amputate at or above the knee in every case in which the gangrene had extended from the toes to the dorsum of the foot.

It is worthy of notice that in Dr. Power's case, although all the conditions were adverse, he still obtained good union in the flaps.—*Dr. C. A. Powers in American Journal of Medical Science.*

---

### ACUTE LARYNGITIS IN CHILDREN.

℞. Ammon. muriat., grs. xvi.
Ext. prun. virg., fl. ʒjss.
Aq. menth. pip., q. s., ad. ℥ij.
M. Sig.—Teaspoonful every two hours.

—*Lancet Clinic.*

### THE EARLY EXTIRPATION OF TUMORS.

In a paper read before the New York State Medical Association, at its recent meeting, Prof. J. W. S. Gouley presented the following conclusions on this subject:

1. Malignant tumors exceed benign tumors in frequency.

2. The malignant tumors comprise epitheliomata, sarcomata and internal adenomata.

3. Among the benign tumors myxomata and external adenomata often recur after incision, but do not infect the system.

4. There is no solid benign tumor that may not become malignant.

5. No means are known by which can be ascertained the precise time of the beginning of metamorphic action in tumors.

6. Most malignant tumors have a stage of benignity.

7. Excision of potentially malignant tumors in the early epoch of their stage of benignity is likely to effect a permanent cure, or at least to prolong greatly the period of immunity from recurrence of the disease.

8. In the excision of malignant tumors the greatest care should be taken to remove as much of the ambient tissues, including fasciæ and lymph glands, as is compatible with good judgment.

9. General treatment of tumors has no value except as an adjuvant of a surgical operation, and is often indirectly injurious, leading the patient to expect a cure by persevering in the use of drugs, and thus allowing the disease to make rapid progress toward a fatal end.

10. Local treatment of tumors, by means of escharotic plasters, pastes or powders, is the most fruitful in evil of all the devices for the torture of the afflicted. The plaster, paste or powder causes the greater part of the tumor to slough, but there is enough left behind for the most rapid extension of the disease. The effect of the escharotic is, therefore, only to till a soil where new growths sprout like so many seeds cast upon rich loam.

11. Compression is delusive in the case of tumors containing cysts, and is directly hurtful by exciting the rapid growth of most tumors.

12. Expectancy, even in the case of benign tumors, is as unwise as meddlesome medication.

13. There should be no waste of time in endeavoring to make a precise diagnosis of a particular morbid growth, for after its excision the microscope reveals the nature of its constituent elements and assists in the establishment of the prognosis, which is the question of greatest importance to the sufferer.

14. What is known of the great fatality of tumors of long standing should induce surgeons to advise the complete removal of all accessible morbid growths as soon as detected, no matter how seemingly trivial or harmless, such as small glandular, fatty, fibrous, and vascular tumors, wens, warts, moles, etc.

15. As soon after excision and as often as a tumor recurs it should be removed, so long as there is any possibility of insuring cicatrization of the wound, even by skin-grafting.

16. Medicinal treatment after the excision of malignant tumors is of much value, even if it consists only in the administration of reconstituent medicines.—*N. Y. Med. Jour.*, Nov. 26, 1892.

---

### WHAT EFFECTS DOES SYPHILIS EXERT UPON THE DURATION OF LIFE?

Dr. Oscar Rogers, according to the *Weekly Medical Review*, discussed this subject with life insurance statistics as a basis. Out of three or four hundred thousand lives insured which he had been enabled to study, there had been fourteen to fifteen hundred syphilitics who had been cured from four to five years. It would require about ten thousand cases as a basis for conclusions which could be regarded as at all reliable. Out of the fourteen to fifteen hundred the mortality had been thirty-seven, which was rather high. The age at death of insured people for a considerable number was about forty-eight, whereas for these syphilitics it had been about forty-one. In other words, syphilitics had apparently lost about seven years of their life by the fact that they had syphilis although they had been "cured" for five years, at the age of thirty the probable length of their lives being instead of sixty-five, fifty-eight; in other words, a loss of one-fifth of their longevity. The man of fifty years had lost one-third of his longevity, the probability being that he would live to only sixty-four instead of to seventy one. Roughly speaking, the syphilitic had probably lost from one-third to one-fourth of his longevity.

Among the 17,000 deaths which he had studied among all classes of insured, he had found that 2 in 170 had died of paresis: whereas, among the syphilitic deaths, paresis was the cause in 1 out of 18. He had found nothing to show that syphilitics were more likely to be carried off by lung disease. He hoped other physicians would add to these statistics.

Dr. Rogers thought that the mortality from syphilis was greatest in continental Europe, then in England, then in the colonies, lastly in the United States. This was, he supposed, to be accounted for, either by the fact that syphilis was milder here, or else we knew better how to treat it.

Dr. Paul Gibier thought it likely the life insurance companies carried many more syphilitics than they supposed, for men did not always consider it obligatory to give a correct answer to interrogatories bearing on this subject. As syphilis is treated to-day, he thought it did not shorten the duration of life. The disease was more severe in new blood.—*Med. Examiner.*

---

### SHOCK AND ITS TREATMENT.

Kottmann (*Wiener Med. Presse*, 1892, No. 21, from Correspondenzblatt für Schweizer Aerzte) states that the most important signs of shock are weakness of the heart, faint respiration, skin pale and covered with cold sweat, face drawn out, sunken eyes, and livid lips; consciousness remains, sensibility reduced. Shock appears when certain very sensitive organs, as testicles, bones, or bowels have been subjected to contusion or other serious injury. Shock may occur from hemorrhage, and is then associated with functional changes in the body of which there are no post mortem evidences. Two theories are advanced: The first defines shock as a reflex paralysis of the heart and vessels, caused by the trauma; according to the second, the injury causes, reflexity, exhaustion of the medulla oblongata and of the spinal cord, and, as the nerve centers are affected, there is debility of heart and respiration. The fever which accompanies shock is, according to Kottmann, the consequence of trauma. The substances causing the fever are here chemical and not bacterial, are freed from normal tissue matter by the injury, and include hemoglobin, fibrin ferment and similar elements. The author objects to confounding shock with debility due to hemorrhage. In the latter there is rapid pulse, quick, deep, panting respiration. In shock, the patient presents the evidence of cerebral irritation, noises in the ears, affected vision, palpitation, yawning, and even convulsions. In severe cases of shock the usual remedies—alcohol, ether, caffeine and camphor—may be used without relief. The similarity in the appearances resulting from hemorrhage and from shock induced Kottmann to use salt water transfusion in four cases with good results. By the neuro-pathological theory the explanation is that the effect of the salt solution is to stimulate the centers with which it comes in contact, and with increased arterial pressure the central nervous system would receive more blood and recuperate more quickly. If the hemo-pathological theory is accepted, the explanation is even more simple; the salt water would fill the empty heart and vessels, and the hydraulic requirements would be fulfilled while the blood in the distended abdominal vessels was being restored to the circulation. Patients suffering from shock often require severe operations, especially the amputation of members. Experience shows that chloroform anæsthesia in these cases is attended with unusual danger; the danger is less with ether, which acts as a heart stimulant.—*American Journal of the Medical Sciences, November*, 1892.

---

### ALLINGHAM'S OINTMENT FOR HEMORRHOIDS.

℞. Bismuth subnit., ʒj.
Hydrarg, subchlorid, gr. j.
Morphin., gr. iij.
Glycerini, ℥ij.
Vaselini, ʒj.

M. Sig.: Use in pile-pipe.

### DYSPNŒA AND ITS TREATMENT BY DRUGS.

Dr. W. Tennant Gairdner classifies his cases as: (1) Due to hæmatic causes, remedied by iron, arsenic, hygienic conditions, food, exercise, possibly by inhalations of oxygen. (2) Due to pulmonary causes, when expectorants are indicated, stimulating the "scavenger muscles" of the bronchial tubes, avoiding the use of opium. (3) Due to extrapulmonary causes other than hæmatic, as cardiac, pleural, or pericardial effusions; dropsical swellings of all kinds; uræmic, when this origin must be carefully considered; and in general the maxim of Baglivi should be observed, to use diuretics, or, in the failure of these, to employ drastic cathartics, or rarely venesection—*Medical Press*, 1892, No. 2779, p. 131.

Prof. Leech finds that the nitrites will relieve certain forms of dyspnœa, and his preference is for the nitrites of ethyl and soda, and nitroglycerin, as more effective and of more lasting influence than nitrite of amyl. A teaspoonful of a 3 per cent. solution of nitrite of ethyl is the most convenient form for administering a nitrite in dyspnœa. In case of failure with the nitrites he advises the vapor of ammonia.

Dr. Hanford believes that the dyspnœa of migraine can be speedily relieved by saline aperients.

Dr. Oliver regards the use of morphine to be beneficial in some cases of bronchitis and of emphysema, accompanied by orthopnœa. In uræmic dyspnœa the nitrites were of very great service, but he would limit venesection to those cases in which dyspnœa was dependent upon, or associated with, dilatation of the right heart.

Dr. Wilberforce Smith advises in spasmodic paroxysms, belladonna, preferably a few drops given with little or no water, or administered by a spray-producer ; in congestive cases, the cautious use of the hot-air bath ; in the dyspnœa of anæmia (in girls) the prohibition of corsets.

Frederick Pearse, in acute spasmodic asthma, and in that associated with tumultuous action of the heart, recommends one or two 5-minim doses of Fleming's tincture of aconite.—*British Medical Journal.*

### THE USE OF WATER IN FEVER.

While the medical world is on the move and has done much in the way of discovery of new remedies which lower the temperature of the body in fever, we hear but little of external medication. It is true that the books speak of baths, and the application of lard or oil, the use of which will do much for the relief of the patient by the softening of the surface and nourishing the system, but in order to gain any permanent relief they should be applied at regular intervals of one, two or three hours, as the temperature of the patient may indicate.

There is another remedy, however, perhaps more powerful than any of these, and that is cold water. There is scarcely any form of fever whatever but will be benefited by sponging the patient gently. It may seem cruel at first ; but the benefit is soon so apparent and the use of it so grateful to the patients that they will often ask for its repetition. We all know how refreshing and cooling a drink of water is to us in health, upon a warm day in summer; and yet how much more is it to a patient being burnt up with fever! But what, perhaps, is a better way of application is to fill a vessel with the cold fluid and pour it in a small stream, from a height of one or two feet, upon the wrist of the sick person for five minutes. By so doing you will rapidly reduce the temperature. The weight of water and the evaporation of it tend very much to increase its good effects. The cooling stream directly over the ulnar and radial arteries tends to cool the current of red-hot blood which is passing through them. The coats of the arteries are contracted, which lessens the flow as well as cools the blood. Within the short time of half an hour you will find the temperature sensibly reduced, and by repeating the application when you find the temperature rising you will be pleased to know you have a remedy which is always safe and sure; the patient always expressing himself as feeling very much better, if he is conscious. You will do in a few moments what you cannot do in hours by internal medication. We ask the profession to try it, and in the way we speak.—*Hopkins in Med. Bulletin.*

### THE RELATIONS OF PELVIC DISEASES TO PSYCHICAL DISTURBANCES IN WOMEN.

At the fifth annual meeting of the American Association of Obstetricians and Gynecologists, held at St. Louis, Dr. George H. Rohé, of Catonsville, Md., read a paper on this subject. The author pointed out the frequency with which bodily conditions influenced mental states. Thus, a torpid condition of the intestines, Bright's disease, putrefactive processes in the intestinal canal, etc., might give rise to melancholia and other disorders of the mental functions. It was not irrational to suppose likewise that diseases of the female sexual apparatus would have a not inconsiderable influence in the production or perpetuation of mental disorders. As a contribution to our knowledge of the subject the following report was submitted : In a hospital containing two hundred insane women, thirty-five were subjected to vaginal examination and twenty-six found with evidences of pelvic diseases. In eighteen of these the uterine appendages were removed with the following results : Sixteen recovered from the operation and two died. Of the sixteen recovered, three had been discharged from the hospital completely restored, both physically and mentally. In ten considerable improvement had followed the operation in both physical and mental conditions, and in three the operation was of too recent a date to allow any definite expression of opinion. The mental disorder present in the eighteen cases was melancholia in six cases, simple mania in one, puerperal mania in four, hysterical mania in one, periodic mania in two, hystero-epilepsy with mania in one, and epilepsy with mania in three. The author, basing his opinion upon his experience, concluded as follows : "The facts recorded demonstrate : first, that there is a fruitful field for gynecological work among insane women ; second, that this work is as practicable and can be pursued with as much success in an insane hospital as elsewhere ; and, third, that the results obtained not only encourage us to continue in the work, but require us, in the name of science and humanity, to give to an insane woman the same chance of relief from disease of the ovaries and uterus that a sane woman has."—*N. Y. Med. Jour.*

### PRACTICAL EXPERIENCE IN THERAPEUTICS.

In a late paper before the Louisville Medico-Chirurgical Society Dr. Jno A. Larrabe mentions some of the therapeutic agents which, during the summer months, had been most successful in his hands. In entero-colitic diarrhœa—the so-called "summer complaint" of cities, dependent upon the various micro-organisms, vitiated air and bad food—salol, naphthaline, carbolic acid, calo-in minute doses, and nitrate of silver stood the test.

In gastro-enteritis he found salicylate of bismuth useful and in inflammatory diarrhœas of infants and older children Rochelle and Epsom salts, in acid infusion of roses with small doses of laudanum. In chronic cases, the nitrous acid camphor mixt. (Hope's) did not fail. For gastric fevers so common in children the preparations, ammonia-phenique, and sulpho-phenique, were mel, used with better success than any other treatment.

He also used this treatment very satisfactorily in the exanthemata.

In diphtheria, peroxide of hydrogen and whiskey internally gave him the best results. Disappointment in the use of the peroxide is often due to the poor quality of the drug. It is a very unstable article. It should always be purchased in small amounts protected from the light, by being put up in four-ounce bottles. On coming in contact with the false membrane of the throat a white foamy reaction takes place. It should be freely applied to the throat on mops or by the use of a spray. The crying and resistance of the child favor the distribution of the fluid. In this way the author has treated successfully a great many cases of diphtheria. He believes that the symptomatic use of definite, although often toxical, doses of whiskey, in children even of tender age, to be the surest safeguard against heart failure.

---

### ASEPSIS AND ANTISEPSIS IN THE LYING-IN CHAMBER.

In a very instructive paper Dr. W. W. Potter lays down the following propositions concerning which he states there should be no dispute:

1. Let us begin by making the patient as nearly clean as possible for soap and water to accomplish.

2. Let her, prior to the beginning of labor, have an immersion-bath daily for several days, and with the first manifestations of pains, let her abdomen and genitalia be rendered absolutely aseptic by the further application of germicides in solution, adequate to accomplish the desired end.

3. Let her have a warm vaginal douche, rendered aseptic.

4. Let the lower bowel be thoroughly evacuated by copious lavements of hot water prior to the vaginal bath.

5. Let her bedding be made as pure and clean as careful laundrying can make it.

6. Let her clothing be made equally clean in like manner.

7. Let there be a number of clean bichloride napkins placed in readiness for use.

If all of these injunctions are rigidly enforced, we have done much to lay the foundation for a physiological labor.

The physician and all the attendants must be rendered as scrupulously asceptic as the patient herself. The nurse must be especially trained in the habit of keeping her hands clean. After the first examination, which should be made as carefully and deliberately as possible, the physician should refrain from further examination unless absolutely required.—*Medical News.*

---

### SIMPLE SORE THROAT.

My views, therefore, in regard to the treatment of acute simple sore throat of the varieties alluded to, may be summarized as follows:

1. I believe that in salol we have a remedy which, in the vast majority of cases, will give relief quicker than any other. Occasionally it utterly fails. Where it does so, I have found that iron tincture with potassic chlorate seems to be the best substitute. It is my conviction that this latter combination finds its best field in those patients who have already had many previous attacks, and in which there is more or less of an interstitial deposit of connective tissue in the mucous membrane. Salol is to most patients far more agreeable than sodium salicylate, and vastly more so than the nauseating guaiac.

2. If peri-amygdalar infiltration has already set in, it is an open question in every case as to whether we shall be able to prevent suppuration. An incision is I believe indicated wherever there is engorgement, even though no pus has yet formed. The latter rarely comes before the fourth day. If it is not found no especial discomfort, then or thereafter, results to the patient from the incision, particularly if a little cocaine is used. The incision should be made where the pus is most likely to form, viz.: high up, in front of and above the pillars, far more commonly the anterior.

3. If pus is present, free incision towards the median line is indicated. It should be followed by a hot bicarbonate of soda gargle, together with poultices on the outside.

4. Care should be taken to thoroughly open the bowels with a mercurial and a saline at the commencement of treatment in any case.—*Dr. Jas. Newcomb, in Jour. Am. Med. Association.*

---

### GONORRHŒA IN WOMEN.

Our knowledge of this subject is comparatively new. Before the paper of Nœggerath in 1873, no adequate conception of the disease existed; especially in its relation to endometritis, salpingitis, peritonitis and sterility.

A woman contracted the disease from her husband. The left vulvo-vaginal gland and the cervix were infected. The disease quickly spread to the Fallopian tubes and peritoneum, resulting in the formation of an abscess to the left of the sigmoid flexure and extending into the true pelvis. Both tubes contained pus. These conditions were revealed when the appendages were removed, a month after the appearance of the symptoms and eleven days after the invasion of the peritoneum.

Does gonorrhœal salpingitis ever end by resolution with restoration of function to the Fallopian tube? Upon the answer to this question depends the propriety of palliative or of radical treatment. The known chronicity of the disease, and its rebelliousness to the treatment in accessible regions where drainage is good, argue against a cure in an inaccessible tube where drainage is difficult if not impossible. No case is known in which gonorrhœal salpingitis has perfectly recovered.

Shall both appendages be removed when only one is infected with gonorrhœa? Tait and others have reported cases where death or a second operation was the result of leaving the second appendage when one was removed for inflammation. No rule can be laid down on this question, but at any rate in women who are already the mothers of families or are approaching the menopause, it is best to remove both appendages.—*Abstract of Dr. Noble's paper read before the American Gynecological Society.*

---

### WHEN TO TREPHINE.

Dr. Lanphear (*Kansas City Medical Index*) says we should operate in every case of fracture of the skull with depression, even if there be no symptoms whatever, and that instances of traumatic epilepsy will diminish in proportion to the frequency of operation, no matter how slight may be the depression. In all cases of local injury to the skull, followed by evidence of inflammation of bone or persistent symptoms of brain irritation, or of pus between the bone and dura mater, the trephine should be resorted to. Where after such injury unconsciousness persists for more than an hour, exploratory operation, including opening the skull, should be done. The appearance of stupor some hours after a head injury indicates meningeal hemorrhage, and requires trephining at the point of injury, if this be known, or at a point indicated by cerebral localization, usually the region of the middle meningeal.—*Medical Record.*

---

### OINTMENT FOR ECZEMA.

℞. Oxide of zinc, gr. xv.
Powdered talc,
Vegetable tar,
Vaseline, of each, ʒv.

Sig.—Make into an ointment, and apply to the part morning and night.—*L'Union Médicale.*

### BRIGHT'S DISEASE.

At the Académie de Médecine M. Lancereaux brought up again the treatment of albuminuria. For him, albumen in the urine was only a symptom, and was of itself without gravity, consequently it should not be dreaded so much as the auto-intoxication produced by excrementitial matter, or in other words, uræmia. The means to employ against this complication is to establish the renal function by diuretics, and when these latter have no longer any effect, by drastic purgatives and frictions of the skin. Then when the uræmia has disappeared, an attempt should be made to repair the renal lesions. Iodide of potassium gives good results in the parenchymatous form of nephritis, while tincture of cantharides, six to twelve drops daily, is very succeseful in removing the dropsy in the interstitial form. As to milk, it is indicated whenever symptoms of uræmic intoxication exists. It was said that albuminuria has frequently accompanied diabetes, and such is the case, but under certain circumstances. In the first place there exists three kinds of glycosuria—constitutional or hereditary diabetes, nervous diabetes of Cl. Bernard, and that having a pancreatic origin. In the first kind, albuminuria is almost always present, depending on sclerosis of the renal artery. In that of Cl. Bernard, on the contrary, it is intermittent and is intimately associated with congestion of both the renal and biliary organs. Obesity is favorable also to the development of albuminuria, of which the inciting cause may be found in the emphysema which so often accompanies the plethoric state.

M. G. Sée said that he could not understand the employment of drastic purgatives and cantharides in the treatment of the affection under consideration, as their administration was totally opposed to all that physiology had taught on the malady. The only agents of any value admissible in albuminuria, in his opinion, are the iodides of potassium, calcium, or strontium. As to cardiac albuminuria, it need not be treated.

M. Lancereaux replied, insisting on the value of purgatives, and above all on the cantharides, which gave him wonderful results where the renal epithelium was affected.—*The Medical Press.*

### THERAPEUTICS OF TYPHOID FEVER IN THE PARIS HOSPITALS.

There exists in Paris much divergence of opinion as to the efficacy of cold baths in the treatment of enteric fever. This method is attributed in France to Brand, of Stettin, although it had been advocated nearly a century ago by Currie. Dr. Juhel-Rénoy has just communicated to the Sociéte Médicale des Hôpitaux the result of an inquiry he has conducted into this question. For this purpose he applied to eighty colleagues, forty of whom replied. Of these hospital physicians fourteen disapprove of the cold bath. Twelve, on the contrary, are enthusiastic believers in the method, which they apply indiscriminately to all cases, whether they be light or severe in character. Two believe in tepid baths gradually cooled down; and eight, while approving generally of cold baths, impose certain restrictions. Of 175 cases treated by the adversaries of the method, 25 died, giving a mortality of 14.2 per cent. Thirty-nine cases treated by the method, with restrictions, yielded one death, or 2.56 per cent. In Professor Bouchard's wards, where progressively cooled tepid baths are in fashion, 54 out of 554 cases died, or 9.74 per cent. The advocates of systematic cold baths lost 40 out of 492 cases, or 8.13 per cent. M. Juhel-Rénoy concluded his paper by expressing his opinion that the introduction of balneotherapy into typhoid fever wards had been the means of reducing the mortality from that disease at least five per cent.—*Lancet.*

---

### TOO MANY PHYSICIANS—A REMEDY.

That there are too many men engaged in the practice of medicine is generally admitted. That there are none too many of those fully qualified for their work is also as generally accepted. We cannot wait for the public to be sufficiently educated to discriminate between competent and incompetent service. We must take the advanced ground and be the teachers ourselves. Not only the remedy, but also the hour in which the remedy itself should be applied is at hand. Instead of scores of medical schools opening wide their doors and crying, Come, let them nearly close these entrances and hedge up the way by a careful and discriminating examination. Let the weeding begin now, and let it keep up at regular intervals throughout the college course. First, there should be an improvement in the quality of the instruction imparted; second, the standard for admission should be raised, and third, the requirements for graduation should be far greater than at present. No school need fear for lack of patronage if it takes this advanced ground. The profession will rally to its support. Instances could be given where this has proved to be the case to a most flattering degree. Begin now to lessen the annual output of doctors, and unfair and often disgraceful means of competition will cease. Commercial methods of obtaining a business will be no more and we will be in word and deed a noble profession.—*National Med. Review.*

---

### THE BATH TREATMENT FOR ECZEMA.

Dr. Oscar Lassar thinks that if the old, erroneous idea that all water and bathing is harmful to eczema and other skin diseases could be overcome, the bathing resorts could not hold the patients who would flock to them to be treated for these troubles. He has himself treated much more than ten thousand cases of eczema of various origin, and only once had occasion to interrupt the use of baths or local fomentations on account of a contra-indication. Fresh cases, and cases of years' standing, even those from which old accepted doctrines would keep every drop of water away, yield to his treatment rapidly. He believes that the cleanliness attained is just as valuable an assistant to the dermatologist as to the surgeon. But not by any means would he limit his therapeutics of skin diseases to baths.

After the bath he would use appropriate pastes, powders, salves and oils the more energetically, for the very reason that they would only remain upon the skin until the next bath, after which they would be renewed or replaced by still better ones. He thinks the whole regimen of the bath cure very valuable, and Berlin pipe-water even will be useful in a degree.—*Therap. Gazette.*

